# Autosomal recessive bestrophinopathy combined with neurofibromatosis type 1 in a patient

**DOI:** 10.1186/s12886-023-02905-5

**Published:** 2023-04-11

**Authors:** Bo Zhao, Lian Chen, Peng Zhang, Ke He, Min Lei, Juan Zhang

**Affiliations:** Department of Ophthalmology, Xi’an No.3 Hospital, The Affiliated Hospital of Northwest University, No.10 eastern section of the third fengcheng Road, Xi’an, 710018 China

**Keywords:** Genetic diagnosis, Neurofibromatosis, Bestrophinopathy, BEST1

## Abstract

**Background:**

Neurofibromatosis type 1 (NF1) is a multisystem genetic disorder that may affect multiple systems of the body. Autosomal recessive bestrophinopathy (ARB) is a rare retinal dystrophy caused by autosomal recessively mutations in bestrophin 1 (BEST1) gene. So far, we have not retrieved any case report of the same patient with both NF1 and BEST1 gene mutations.

**Case presentation:**

An 8-year-old female patient with café-au-lait spots, freckling on skin presented to our ophthalmology clinic for routine ophthalmological examination. Her best corrected visual acuity (BCVA) was 20/20 in both eyes. Slit-lamp examination of both eyes revealed few yellowish-brown dome-shaped Lisch nodules over the iris surface. Fundus examination was notable for bilateral confluent yellowish subretinal deposits at macula, few yellow flecks at temporal retina, and cup-to-disc ratio of 0.2. Optical coherence tomography (OCT) revealed subretinal fluid (SRF) involving the fovea, elongated photoreceptor outer segments and mild intraretinal fluid (IRF) at bilateral macula. Fundus autofluorescence demonstrated hyperautofluorescence in the area corresponding to the subretinal deposits. Whole-exome sequencing and Sanger sequencing were used to investigate genetic mutation in the patient and her parents. A BEST1 gene heterozygous missense c.604 C > T (p.Arg202Trp) was identified in the patient and her mother. Also, the patient carries an NF1 nonsense mutation c.6637 C > T (p.Gln2213*) with the mosaic generalized phenotype. There were no visual impairments or obvious neurological, musculoskeletal, behavioral or other symptoms in this patient, so she was managed conservatively and advised to follow up regularly for a long time.

**Conclusions:**

ARB and NF1, which are caused by two different pathogenic gene mutations, have rarely coexisted in the same patient. The discovery of pathogenic gene mutations may play a crucial role in more accurate diagnostics and genetic consultations for individuals and their families.

## Introduction

Neurofibromatosis type 1 (NF1) is an autosomal dominant, multisystem disorder with an incidence of 1:3000 [1]. NF1 is caused by pathogenic variants in NF1 gene on chromosome 17q11.2 and characterized by skin pigmentation anomalies such as café-au-lait spots and freckling, as well as dermal neurofibromas. Additionally, NF1 patients frequently have melanocytic iris hamartomas (Lisch nodules), seizures, neurofibromas, learning disabilities, attention deficits, skeletal abnormalities [2, 3]. According to the diagnostic criteria of NF1 established by the National Institutes of Health Consensus Conference in 1988, patients with 6 or more café-au-lait spots measuring at least 5 mm in size before puberty, axillary or inguinal freckles and Lisch nodules on the iris could be definitively diagnosed without the requirement of any additional tests [4].

Autosomal recessive bestrophinopathy (ARB) is a rare inherited retinal dystrophy defined by Burgess et al. [5] in 2008. ARB is caused by mutations in both alleles of the bestrophin 1 (BEST1) gene with either homozygous or compound heterozygous mutations [6, 7]. BEST1 is an integral membrane protein known to function as a Ca ^2+^ activated and volume-regulated chloride channel localized to the basolateral membrane of retinal pigment epithelium (RPE) [8]. Mutations in BEST1 therefore affect RPE metabolism and cause a collection of bestrophinopathies, such as Best disease [9], adult-onset Best vitelliform macular dystrophy [10], ARB [11], Retinitis pigmentosa 50/concentric retinitis pigmentosa [12], autosomal dominant vitreoretinochoroidopathy (ADVIRC) [13] and microcornea, rod-cone dystrophy, cataract, and posterior staphyloma syndrome (MRCS) [14]. The clinical features of ARB include multifocal vitelliform deposits with subretinal fluid (SRF) and intraretinal fluid (IRF), hypermetropia, and shallow anterior chambers, which are predispositions to narrow-angle glaucoma may co-occur [15].

Here, we present the rare case of NF1 gene and BEST1 gene mutation coexisted in a patient who displayed two typical spectrums of clinical phenotypes corresponding to pathogenic gene mutations.

## Case report

**Fig. 1:**
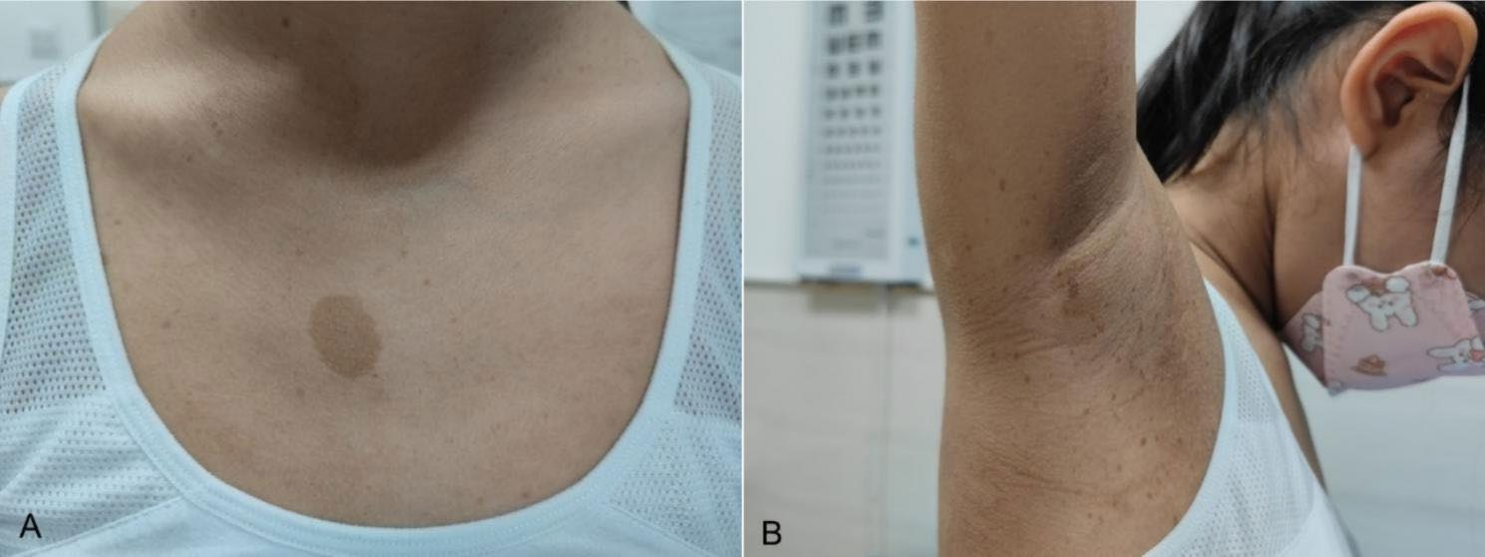
Photos of the patient with NF1. **a** A irregularly shaped café-au-lait spot with sharp border on chest. **b** Freckling spread over the skin of right axillary region

An 8-year-old female presented to our ophthalmology clinic for routine ophthalmological examination. We noticed an irregularly shaped café-au-lait spots with sharp border on her chest (Fig. 1A). Further visual inspection revealed multiple café-au-lait spots on the skin of back, abdoman, bilateral thighs and the back of right hand. Additionally, bilateral inguinal and axillary freckling were also present (Fig. 1B).

**Fig. 2:**
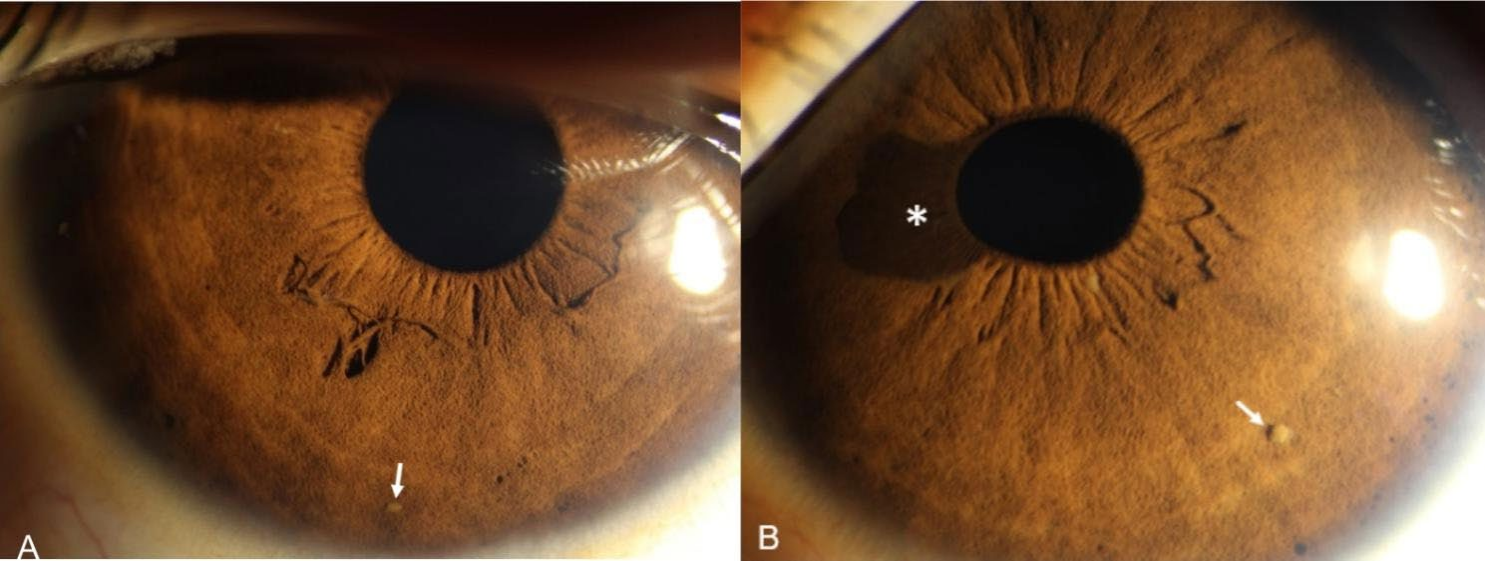
Slit-lamp anterior-segment photography of the irises in patient with NF1. **a** Lisch nodule (white arrow) on the stromal surface of right iris. **b** Lisch nodule (white arrow) on the surface of left iris, and an iris nevus involving the pupil margin (star)

The uncorrected visual acuity (UCVA) in her right eye was 20/32, improving to 20/20 with + 0.5 diopter of spherical (DS)/ -1.50 diopter of cylinder (DC) at 170°. The left eye UCVA was 20/25, improving to 20/20 with + 0.50 DS/-1.50 DC at 175°. Ocular biometry readings showed an axial length (AL) of 22.62 mm in the right eye and 22.66 mm in the left eye, and intraocular pressure (IOP) was 12 mmHg and 14 mmHg in the right and left eye respectively. Slit-lamp examination of both eyes revealed few yellowish-brown dome-shaped Lisch nodules over the iris surface (Fig. 2). In left eye, a brown, slightly elevated iris nevus about 4 mm in diameter was noticed (Fig. 2B). The anterior chamber angle of each quadrant in both eyes was normal.

**Fig. 3:**
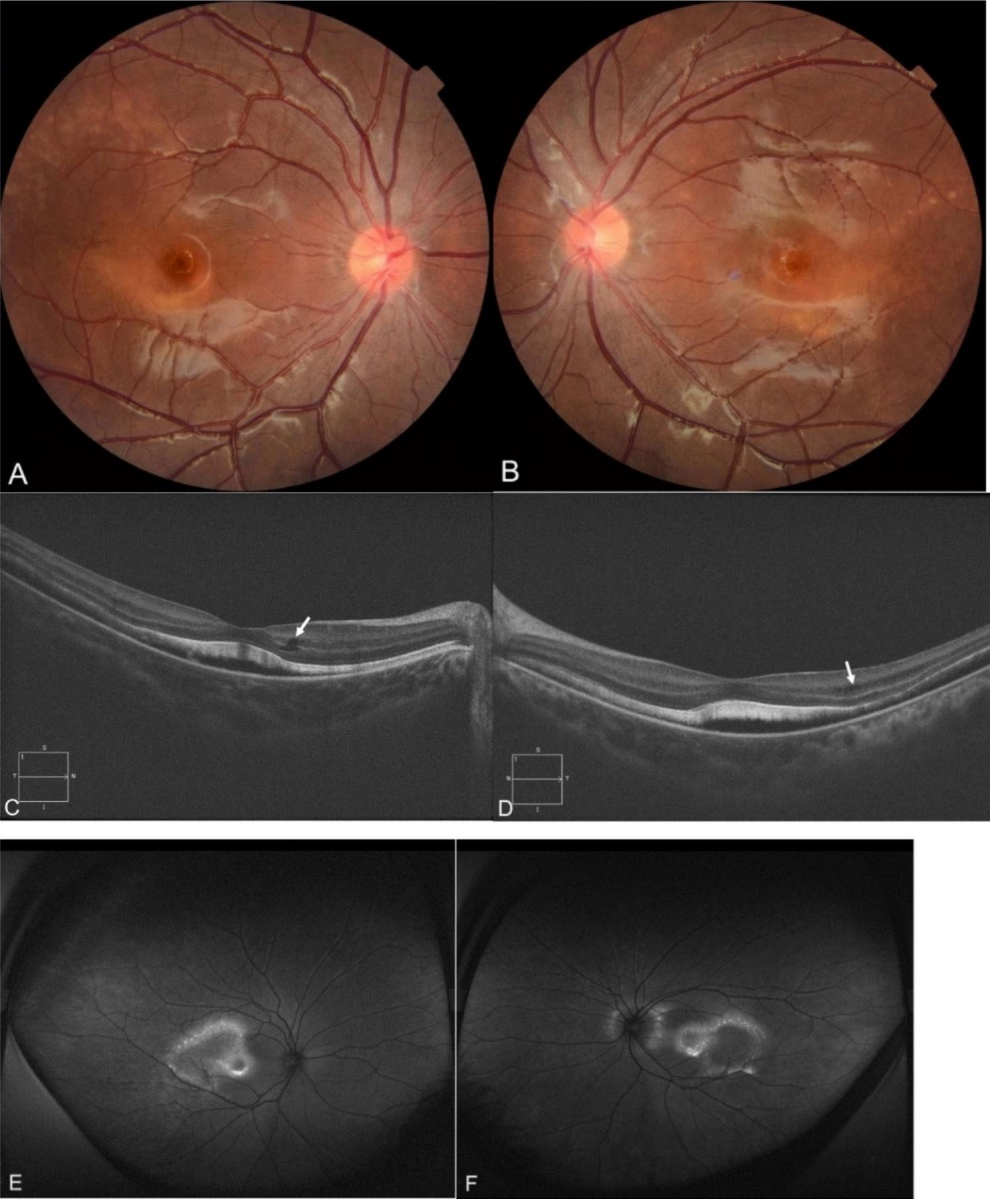
Multimodal fundus imaging of a patient with NF1. **a, b** Color fundus photographs of right eye and left eye showing confluent yellowish subretinal deposits at macula, few yellow flecks at temporal retina. **c,d** OCT of right eye and left eye showing subretinal fluid involving the fovea, elongated photoreceptor outer segments and intraretinal fluid (white arrow) at macula. **e,f** Fundus autofluorescence of right eye and left eye showing hyperautofluorescence located in the macula and its temporal side, which corresponding to the location of subretinal deposits

Anterior segment of both eyes had deep anterior chamber and clear lens. Fundus examination was notable for bilateral confluent yellowish subretinal deposits at macula, few yellow flecks at temporal retina, and cup-to-disc ratio of 0.2 (Fig. 3A and B ).

Optical coherence tomography (OCT) revealed a marked amount of SRF involving the fovea, elongated photoreceptor outer segments and mild IRF at bilateral macula (Fig. 3C and D). Fundus autofluorescence demonstrated hyperautofluorescence in the area corresponding to the subretinal deposits (Fig. 3E and F) .

**Fig. 4:**
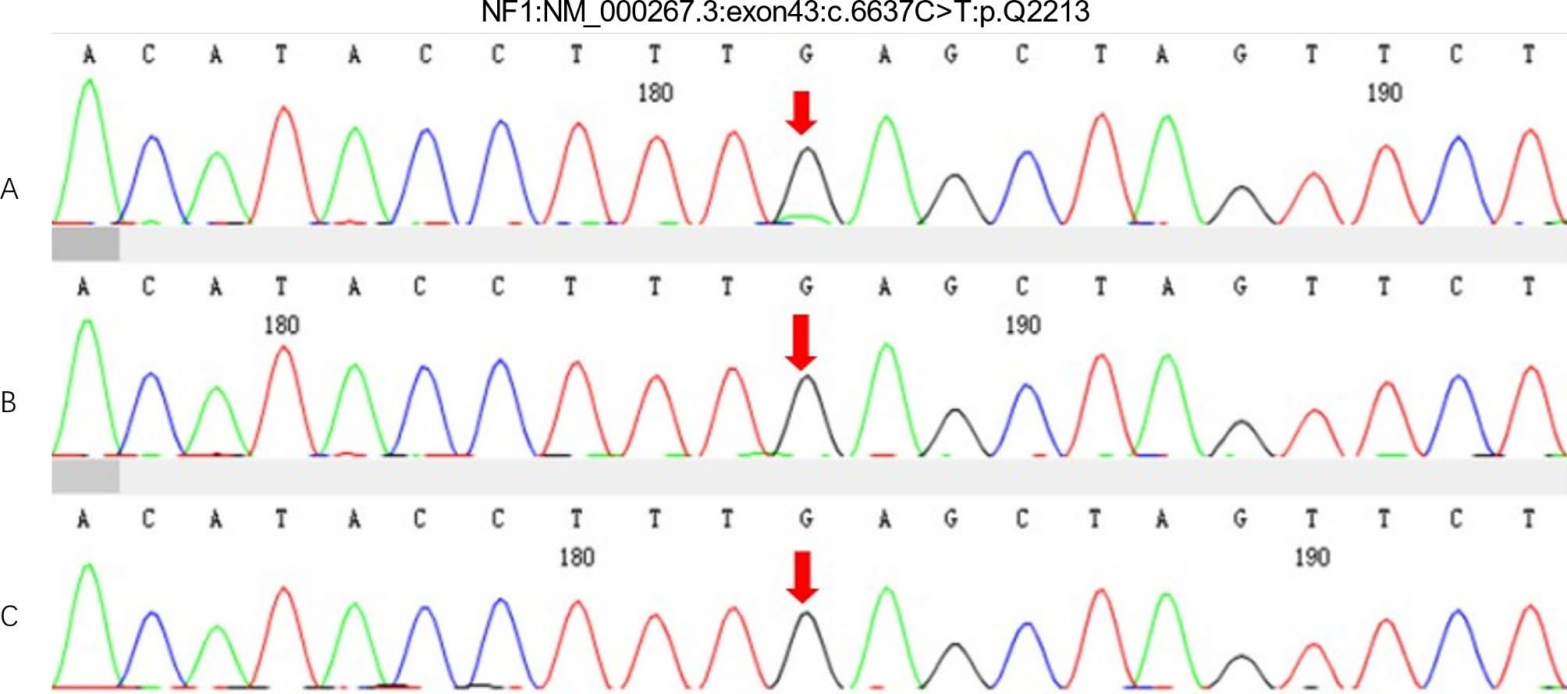
Whole-exome sequencing of the patient and her parent. **a** A mosaic nonsense NF1 gene mutation c.6637 C > T (p. Gln2213 *) were dectected in the patient. **b** No variation were detected in the patient’s father. **c** No variation were detected in the patient’s mother

**Fig. 5:**
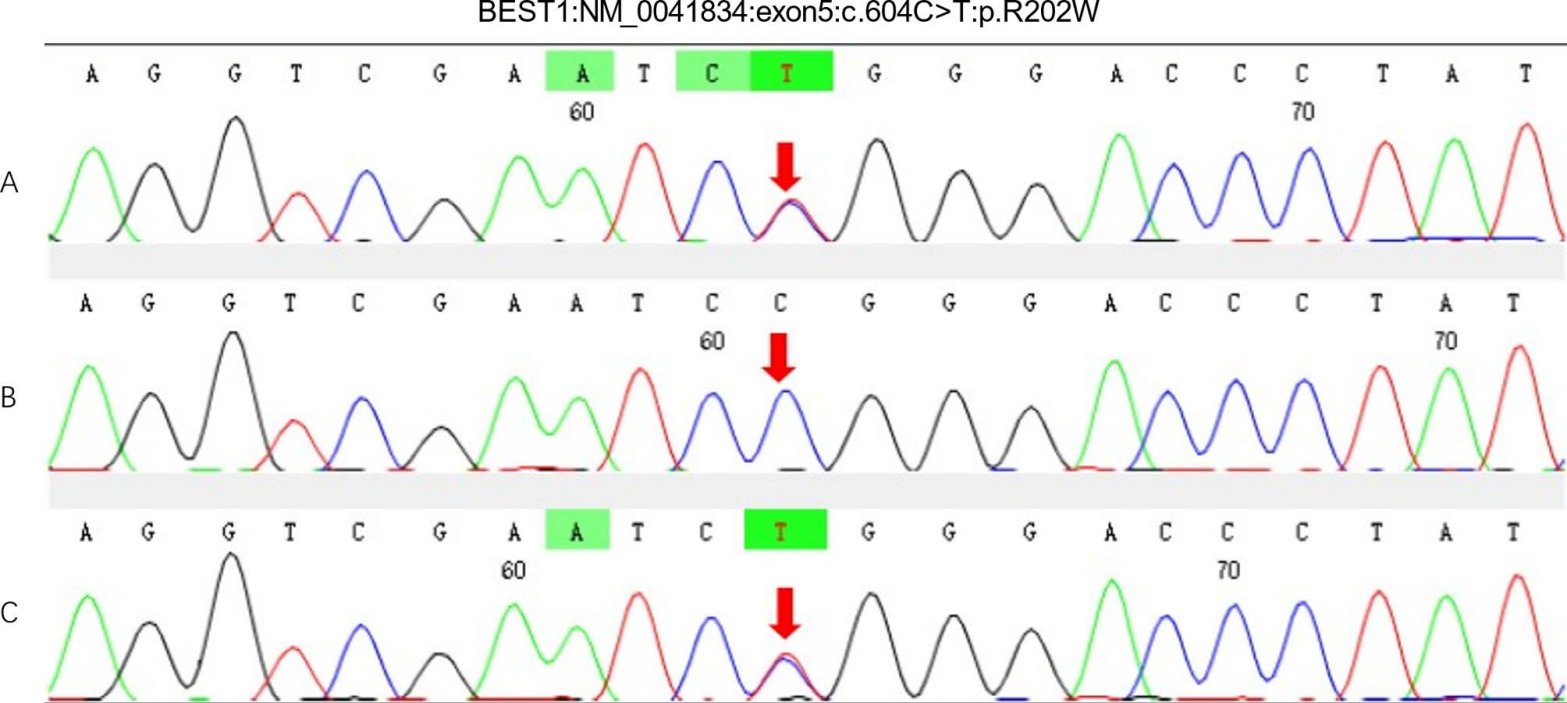
Whole-exome sequencing of the patient and her parent. **a** A heterozygous missense BEST1 mutation c.604 C > T (p. Arg202Trp) were dectected in the patient. **b** No variation were detected in the patient’s father. **c** The same heterozygous mutation of c.604 C > T were dectected in the patient’s mother

Subsequently, blood samples were collected to investigate genetic mutation in the patient and her parents by whole-exome sequencing and Sanger sequencing validation. A NF1 nonsense mutation c.6637 C > T (p. Gln2213*) with the mosaic generalized phenotype in the patient was found, this mutation was not detected in her parents (Fig. 4). A bestrophin 1 (BEST1) gene heterozygous missense variation c.604 C > T(p. Arg202Trp) was identified in the patient and her mother (Fig. 5) .

Magnetic resonance imaging (MRI) scan of the patient’s brain did not reveal any obvious structural abnormalities. Her parents are not consanguineous and reported that the Café-au-lait spots and frickling on the patient’s skin had existed since birth and the number gradually increased with age. Finally, the patient was diagnosed with NF1, and ARB. Her parents also received a comprehensive ophthalmological examination, but no abnormality was found.

Because the patient had no visual impairment, the patient has been healthy since childhood and has no obvious neurological, musculoskeletal, behavioral as well as other symptoms, she was managed conservatively and advised to follow up regularly for a long time.

## Discussion

In this study, we reported a rare case carries NF1 nonsense mutation c.6637 C > T (p. Gln2213*) and BEST1 gene heterozygous missense variation c.604 C > T(p. Arg202Trp), which has not been reported before.

Although mutation c.6637 C > T (p.Gln2213*) has not been reported before, the diagnosis of NF1 can be determined according to diagnostic criteria based on Café-au-lait spots, iris Lisch nodules and axillary freckling in this case. Neither her parents nor members of her extended family had any features of neurofibromatosis, and mutation c.6637 C > T (p. Gln2213 *) did not detected in her parents. Therefore, we assumed that the mutation of NF1 gene has occurred in early embryogenesis of the patient, which in turn has led to a mosaic generalized phenotype of NF1.

However, ARB and NF1 are two distinct genetic disorders. Compound heterozygous or homozygous mutations of the BEST1 gene is responsible for the ARB, which is usually inherited in an autosomal recessive pattern [16]. Unlike other early onset retinal dystrophies, children with ARB typically present good central vision in the first decade of life [17]. Progressive deterioration of visual function was identified in older patients (> 18 years of age) and in those with longer follow-up (≥ 5 years). In addition to the retinopathy, ARB is strongly associated with abnormal iridocorneal anatomic features, shallow anterior chamber depth, and reduced axial length and increased susceptibility to angle-closure glaucoma [17]. Xuan et al. [18] claimed that about 50% of ARB patients had angle closure glaucoma, which was based on the study of the clinical and genetic characteristics of a large cohort of Chinese patients with vitelliform macular dystrophies.

Identification of the mutation in the BEST1 gene is essential for the definitive diagnosis of ARB. To date, over 370 variants have been identified among BEST1-associated retinopathies [19]. In our case, whole-exome sequencing revealed c.604 C > T (p.Arg202Trp) combined heterozygote mutation. Mutation c.604 C > T (p.Arg202Trp) in Best1 has been reported in ARB and BVMD [20, 21].

BEST1 gene encodes the 585-amino acid protein bestrophin-1 (Best1), is expressed predominantly in the RPE and localizes mostly to the basolateral plasma membrane of the cells [22]. Best1 functions as both an anion channel and a regulator of intracellular Ca^2+^ signaling in RPE [23, 24]. Mutations in BEST1 may lead to dysregulation of the anion channel in RPE, subsequently resulting in defective fluid transport in RPE, accumulation of fluid in the subretinal space and impaired ability to phagocytose photoreceptor outer segments (OS), which characterized by elongated photoreceptor OS that are uneven in length as well as piles of debris in the subretinal space [25, 26].

The accumulation of lipofuscin in the RPE has been associated with the development of retinal diseases, particularly age-related macular degeneration and Stargardt disease. A major component of lipofuscin is the bis-retinoid N-retinylidene-N-retinylethanolamine (A2E). The incomplete lysosomal degradation of photoreceptor OS debris resulting from the phagocytosis of RPE cells formed heterogeneous lipofuscin granules (LGs) [27]. The A2E, photooxidation and photodegradation products of bisretinoids in LGs can emit fluorescence excited by a wavelength of 488 nm [27, 28].

The heterozygote mutation c.604 C > T (p.Arg202Trp) was also detected in patient’s mother, indicating that the variation is inherited from her mother. Since the juvenile patient’s vision is normal, and there are no other abnormalities except skin pigmentation, iris Lisch nodules and retinopathy, the systemic or local lesions caused by these two gene mutations may still occur or worsen with age, such as angle-closure glaucoma. We suggest that the patient should be closely followed up during whole life time and managed conservatively at present.

## Conclusion

This is the first case reported in literature in which a patient with NF1 and ARB concurrently, caused by NF1 and BEST1 gene mutations respectively. Identification of pathogenic gene mutations is likely to contribute significantly to understanding the pathogenesis and genetic consultation of individuals and their family members.

## Data Availability

The clinical data used in the current study are available from the corresponding author on reasonable request.

## Abbreviations

NF-1: Neurofibromatosis type 1
ARB: Autosomal recessive bestrophinopathy
BEST1: Bestrophin 1
IRF: Intraretinal fluid
SRF: Subretinal fluid
RPE: Retinal pigment epithelium
ADVIRC: Autosomal dominant vitreoretinochoroidopathy
MRCS: Microcornea, rod-cone dystrophy, cataract, and posterior staphyloma syndrome
BCVA: Best corrected visual acuity
IOP: Intraocular pressure
OCT: Optical coherence tomography
MRI: Magnetic resonance imaging
A2E: N-retinylidene-N-retinylethanolamine
OS: Outer segment
LG: Lipofuscin granule
